# Hypersensitive pupillary light reflex in infants at risk for autism

**DOI:** 10.1186/s13229-015-0011-6

**Published:** 2015-03-03

**Authors:** Pär Nyström, Gustaf Gredebäck, Sven Bölte, Terje Falck-Ytter, EASE team

**Affiliations:** Uppsala Child & Babylab, Department of Psychology, Uppsala University, Uppsala, Sweden; Center of Neurodevelopmental Disorders at Karolinska Institutet (KIND), Pediatric Neuropsychiatry Unit, Department of Women’s & Children’s Health, Karolinska Institutet, Stockholm, Sweden; Division of Child and Adolescent Psychiatry, Stockholm County Council, Stockholm, Sweden

## Abstract

**Background:**

*Post mortem* brain tissue data and animal modeling work indicate cholinergic disruptions in autism. Moreover, the cholinergic system plays a key role in the early neurodevelopmental processes believed to be derailed early in life in individuals with the disorder. Yet, there is no data from human infants supporting a developmentally important role of this neurotransmitter system. Because the pupillary light reflex depends largely on cholinergic synaptic transmission, we assessed this reflex in a sample of infants at risk for autism as well as infants at low (average) risk.

**Methods:**

Ten-month-old infants with an older sibling with autism (*n* = 29, 16 females), and thus a genetic predisposition to developing the disorder themselves, were presented with white flashes on a computer monitor, and pupillary responses were captured using eye tracking. A control group matched on age and developmental level (*n* = 15, seven females) was also tested.

**Results:**

The siblings of children with autism had a faster and stronger pupillary light reflex compared to control infants. Baseline pupil diameter was equal in the two groups, ruling out tonic autonomic imbalance as an explanation for these differences.

**Conclusions:**

This study establishes that infant siblings of children with autism have hypersensitive pupillary light reflexes, a result which supports the view that altered sensory processing in infancy is associated with elevated autism risk. Moreover, the study indicates that individual differences in autism susceptibility are linked to differences in the cholinergic system during an early developmental period.

## Background

Autism spectrum disorder (herein termed autism) is characterized by early emerging social communication and interaction deficits alongside restricted, repetitive patterns of behaviors and interests. It is a heterogeneous and common neurodevelopmental disorder and poses substantial challenges to the individual and the family as well as the wider society. Little is known about the early neurodevelopmental events that shape the autistic behavioral phenotype, which typically emerges during toddlerhood and early childhood [1]. Therefore, many studies are currently being carried out on infants at risk for autism (for example, siblings of children with the disorder, in which the recurrence rate may be as high as 20%) which can inform about processes in infancy that are associated with autism susceptibility [2,3].

Here, we tested the hypothesis that the pupillary light reflex is altered in infants at risk for autism. There were several reasons for this focus. First, the pupillary light reflex regulates the amount of light that reaches the retina, and recent theories of autism as well as the latest edition of the Diagnostic and Statistical Manual of Mental Disorders (DSM-5) view altered reactivity to sensory input as a core defining feature of the disorder [4,5]. Second, although no data on the pupillary light reflex are available from infants, a study of adults with autism found strikingly reduced pupillary light reflex compared to controls [6]. Finally, autism research is in strong need for biological markers that can inform the search for precise molecular mechanisms and that have high translational potential [7]. The pupillary light reflex is noninvasively assessable and is mediated by a well-characterized and relatively simple neural circuit [8]. Specifically, the reflex arc consists of four neurons, bypassing the thalamus and cortex. It depends on synaptic transmission in the Edinger-Westphal nucleus and the ciliary ganglia, both of which are highly acetylcholine-dependent [8,9]. For this reason, the pupillary light reflex has been seen as an index of the integrity of the cholinergic system in other brain disorders [10]. The cholinergic system plays a key role in the pre- and postnatal neurodevelopmental processes believed to be derailed early in life in individuals with autism, including neuronal pathfinding and cell survival [11,12]. Neuroimaging findings as well as *post mortem* brain tissue data [13-15], rodent models [16,17], and molecular genetic studies [18] suggest that alterations of the cholinergic system are involved in the etiology of autism. Critically, however, there is no data from infants available to support this hypothesis.

A demonstration that infants at risk for autism have an altered pupillary light reflex would add credibility to the view that individual differences in autism susceptibility early in life are linked to the development of the cholinergic system. In this study, we report the relative constriction of the pupil and the latency to constriction onset in a group of infants at risk and in a control group.

## Methods

### Participants

We included a group of 10-month-old infants with an older full sibling with autism (*n* = 29, 16 girls). We also included a group of low (average)-risk infants (*n* = 15, seven girls). The two groups were well matched in terms of chronological and developmental age (cognitive and motor skills) and socioeconomic background (Table 1). The participants were part of an ongoing longitudinal study that follows infants from 10 months to 3 years of age. Here, data from the first assessment (10 months) are reported. High-risk infants were recruited through the project’s web site, advertisements, and clinical units. Low-risk infants were recruited from live birth records. Both groups were primarily from the larger Stockholm area. High-risk infants all had at least one older sibling with a community diagnosis of autism spectrum disorder in accord with current regional guidelines which include first-choice standardized diagnostic instruments. Medical records for the older sibling were collected to confirm the diagnostic status. In our project, we could confirm the use of the ADOS or the ADI-R in 70% to 75% of the older siblings through inspection of the obtained medical records, and because details about specific instruments are not always included in the records, the actual figure is likely to be higher. Low-risk infants had no relatives (up to second degree) with autism. To match the high-risk group, we required that all infants in the low-risk group also have at least one typically developing older sibling. No infant from either group was born prematurely (<36 weeks) or had known/suspected medical or developmental concerns, including visual/auditory impairments. The Mullen Scales of Early Learning [19] were conducted with each infant by an experienced licensed psychologist to assess the infants’ developmental level. More than 90% of infants in both groups had parents that were born in Sweden. Another 18 infants were tested but excluded from the statistical analysis. Six of these were excluded from statistical analysis due to being half siblings (high-risk: *n* = 5; low-risk: *n* = 1). In addition, 11 infants were excluded due to insufficient number of valid trials (high-risk: *n* = 9; low-risk: *n* = 2). Video recordings of the infants during the eye-tracking session indicated that excess movement contributed to data loss in seven of these cases (six high-risk and one low-risk) and incorrect positioning of the infant relative to the eye tracker in three cases (two high-risk and one low-risk). In one case, the video recording was not available due to technical problems. This infant had large amounts of missing and noisy data during the whole eye-tracking session. Finally, one high-risk infant contributed with enough trials but was excluded due to extreme relative amplitude values (>3 std from the high-risk group and >2 std from the low-risk group).

**Table 1:** **Study group characteristics**, **final samples** (**mean**/**standard deviation**)

**Measure**	**HR** ***n*** **= 29**	**LR** ***n*** **= 15**	**Pairwise comparison (** ***P*** **value** ^**a**^ **)**
Age (days)	312/15	313/14	.807
MSEL^b^ total score	99/13	98/11	.887
MSEL VR^c^	53/9	54/9	.738
MSEL FM^d^	56/10	55/10	.720
MSEL RL^e^	45/11	44/11	.911
MSEL EL^f^	43/10	43/10	.996
SES^g^	−0.1/1.0	0.1/1.0	.598
Eye color^h^	1.5/1.0	1.3/0.9	.656

^a^Independent samples *t*-test; ^b^Mullen Scales of Early Learning (MSEL); ^c^visual reception (VR) subscale; ^d^fine motor (FM) subscale; ^e^receptive language (RL) subscale; ^f^expressive language (EL) subscale; ^g^socioeconomic status (SES), calculated on the basis of parental education and income (equal weight), expressed as *z*-score; ^h^Experimenter rated (0 to 3, 3 = darkest); high risk (HR); low risk (LR).

### Data collection and analysis

**Figure 1:**
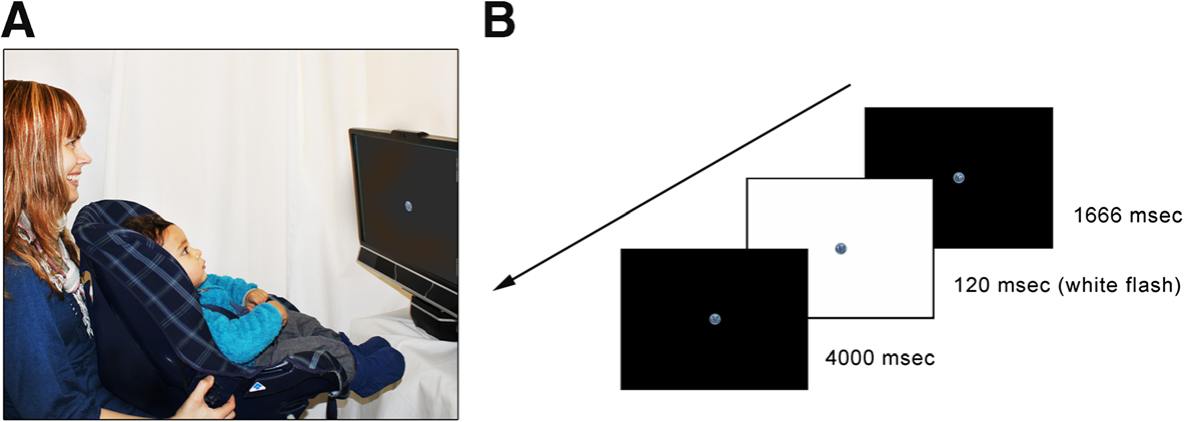
**Experimental setup and stimuli. (A)** Pupillary light reflex measured using noninvasive eye-tracking technology (picture published with consent). **(B)** Brief flashes of light were shown on monitor, while eye tracker measured induced changes in pupil size.

Noninvasive eye-tracking technology suitable for infant populations (Figure 1A) was used to measure pupil changes in response to brief flashes of light. Pupil data were collected by a Tobii 1750 eye tracker (Tobii Technology, Danderyd, Sweden) with a sampling rate of 50 Hz in a room with a controlled ambient light level of 0.9 lux. Each stimulus lasted approximately 6 s and consisted of a small central fixation point on a black background (0.9 lux) that flashed white (190 lux) for 75 ms, that is, substantially above visual thresholds [8], with a random onset between 1,600 and 2,400 ms (Figure 1B). The stimulus was presented 16 times to each infant and interleaved with other video clips with a total session duration of approximately 7 min (no breaks). Before each trial, a 15-s video clip with two dynamically moving point light displays (16 points) was presented, one on each side of the screen [20]. This bilateral presentation caused a saccade from the side of the screen to the center of the screen at every trial onset, thereby reducing the risk of different retinal saturation between infants.

Computational analysis was done in MATLAB (The MathWorks, Inc., [21]) using the TimeStudio framework [22]. Gaps in the data series (maximum two samples) were linearly interpolated, and trials with <90% looking time during the time interval 0- to 1,000-ms post flash were discarded. This conservative criterion ensured that eye blinks (which produce >100-ms data loss) were not influencing the pupil measures. Infants with less than four valid trials were excluded from further study (*n* = 11, see above for details). Pupillary light reflex latencies were calculated according to established standards [23]: a five-point second-order polynomial moving Savitzky-Golay filter was applied before resampling the signal to 300 Hz to achieve better temporal resolution. The velocity was calculated by taking the derivative of the pupil-size signal, and the acceleration was calculated by taking the derivative of the velocity. A centered 25-point moving average filter was applied to the pupil size, velocity, and acceleration traces before further processing. The latency was defined as the absolute acceleration maximum in the interval 100 to 500 ms for each trial, and the median latency across trials was used as the dependent measure in final statistical analyses.

**Figure 2:**
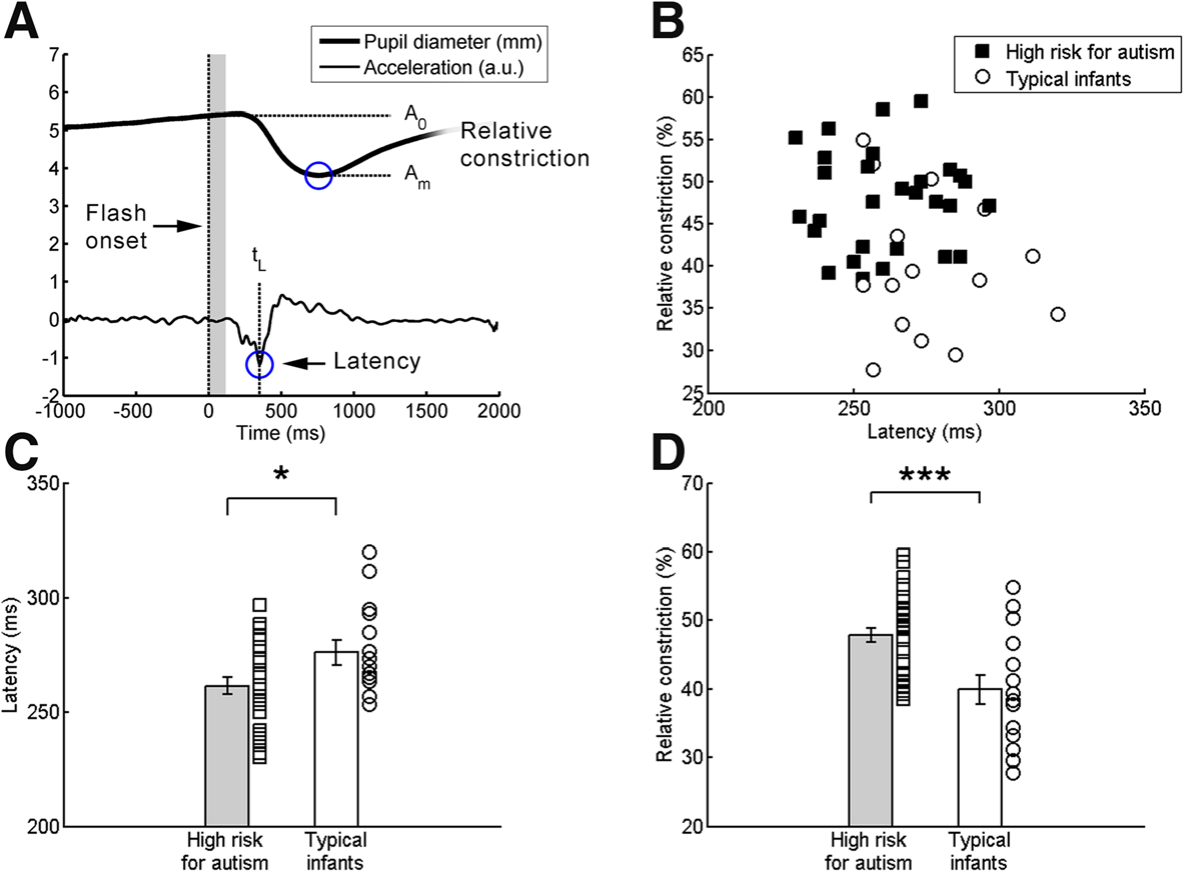
**Stronger and faster pupillary light reflexes in infants at risk for autism. (A)** Relative pupil constriction, (A_0_^2^ − Am^2^)/A_0_^2^, was calculated for individual trials. Latency was determined from the minima of the second derivative of the pupil-size time series (maximum constriction acceleration). Traces show actual data from one trial from one infant. **(B)** Scatterplot showing individual data for both measures. **(C, D)** Groups differed in both latency and constriction amplitude. *P < .05, ***P < .005; error bars show standard error of the mean.

The relative pupil constriction was calculated as in Fan et al. [6] by the formula (A_0_^2^ − A_m_^2^)/A_0_^2^, where A_0_ is the average pupil diameter before onset of pupillary light reflex (in the time interval 0 to 120 ms) and A_m_ is the minimum pupil diameter in the interval 0 to 1,500 ms relative to the white flash (Figure 2A).

### Statistics

Analyses of the data distributions confirmed the normality and homogeneity of variance. We used statistical tests (independent *t*-test) with two-tailed probabilities (*α* = 0.05) unless otherwise stated. Group comparisons were performed using the independent *t*-test with bootstrapping (10,000 permutations) to obtain robust confidence intervals (running the analyses with conventional *t*-tests yielded the same pattern as reported below but with lower *P* values (amplitude *P* < .001; latency *P* = .025)).

## Results

The relative constriction in response to the light flashes was larger in high-risk (mean 47.84%, standard deviation (SD) 5.84%) than in low-risk (mean 39.89%, SD 8.29%) infants (*t*(42) = 3.70, *P* = .003). Moreover, the latency of the reflex onset was significantly shorter in high-risk (mean 261.38 ms, SD 19.27 ms) than in low-risk (mean 276.00 ms, SD 20.94 s) infants (*t*(42) = 2.32, *P* = .029). Thus, both measures demonstrated hypersensitive pupillary light reflexes in the high-risk group. Pupil data for the two groups are shown in Figure 2.

There were no differences between the high-risk and low-risk groups in percent looking time on the screen during a 10-s interval before the flash (*t*(42) = .97, *P* = .34), showing that the difference in the pupillary response cannot be explained by different saturation of the retina in the two groups. Also, there were no correlations between the percentage looking and the pupillary light reflex measures. Neither the correlation between looking time and relative constriction (*r*(42) = .09, *P* = .570, Pearson correlation) nor the correlation between looking time and latency (*r*(42) = −.02, *P* = .900) was significant.

Tonic autonomic imbalance would be expected to cause altered pupil diameter at baseline, but not any difference in the latency of the pupillary light reflex [8,9]. Baseline (A_0_) pupil diameter was similar between the two groups (high-risk, mean 4.80 mm and SD 0.56 mm; low-risk, mean 4.68 mm and SD 0.54; *t*(42) = 0.66, *P* = .498). Thus, the current results cannot be explained by tonic autonomic imbalance in the high-risk group.

## Discussion

This study shows that infants at risk for autism have a hypersensitive pupillary light reflex. Thus, at a general level, the study supports theories emphasizing sensory abnormalities as well as the inclusion of sensory hypo- or hyper-reactivity in the diagnostic criteria for autism [4,5]. Moreover, given the link between the pupillary light reflex and the cholinergic system [8-10], the study also provides specific, albeit indirect, support for the view that cholinergic disruptions could be involved in the etiology of the disorder. The detection of these group differences prior to the emergence of the symptoms of autism [24] adds credibility to the view that cholinergic disruption may have a causal role, as hypothesized earlier based on nondevelopmental human data and animal work [13-16,18].

It is notable that the direction of the group difference is opposite to what could be expected from one study of the pupillary light reflex in adults, which showed hyposensitivity to light in individuals with autism [6]. However, a recent study that included children aged 6 years and older indicated that the magnitude of the difference (autism vs. typical) was smaller (and absent for some measures) in the youngest children [25]. This finding, together with the current data, indicates that chronological age is an important factor when interpreting the pupillary light reflex effect in relation to autism risk. Reversal of group differences as a function of time has been reported by at least two recent studies comparing autistic infants with controls [26,27].

Recent studies indicate that shared environmental influences are negligible in autism, and the genetic risk is predominantly due to common variants ([28,29] but see [30]). If correct, studies of infant siblings primarily shed light on the early processes associated with genetically mediated autism risk. The recurrence rate in infant siblings of children with autism has been estimated to be around 20% based on US data [3]. A European study of siblings reported that more than 30% received an autism diagnosis at follow-up [31]. In addition, a substantial portion of the siblings are expected to develop other significant sociocommunicative, cognitive, or motor problems or delays. Together, this means that about 50% of siblings of children with autism will eventually have significant autism-related developmental concerns [24].

It is important to note that without follow-up data, the relation between the data presented here and later autism diagnosis is not known. Rather than predicting autism *per se*, the pupil response could be a part of the early broader autism endophenotype - shared between both affected and unaffected individuals in the high-risk group. Although identifying such endophenotypes is of lower immediate clinical value, it is important for our understanding of the biological processes associated with susceptibility for disease. It is interesting to note that the latency and the amplitude data were unrelated (Figure 2B), but that both measures differed between groups (Figure 2C,D). Thus, the two could reflect separate underlying processes, which may in turn be differentially related to developmental outcomes.

Given the relative simplicity of the reflex arc, the demonstration of an altered pupillary light reflex in infants at risk for autism could help narrow the search for biological mechanisms related to the disorder [8]. The fact that the reflex can be studied in animals adds to this argument. It is notable that the nicotinic acetylcholine receptor subunit alpha 7 is upregulated in individuals with autism [14] and mediates fast excitatory synaptic transmission in the pupillary light reflex circuit in animals [32]. Thus, although speculative, one hypothesis arising from the current work is that the fast response in the high-risk group is linked to acetylcholine nicotinic receptors, which are known to modulate other autism-related neurotransmitters as well [11,33]. Future studies should also evaluate whether retinal [34] or axonal [26] differences could be involved in shaping the altered pupil response in the infants at risk for autism.

## Conclusions

That infants at risk for autism have hypersensitive pupillary light reflexes provides support for the view that early cholinergic disruptions could play a role in the etiology of autism and related disorders of development. In addition, the current study suggests that pupillometry could contribute to noninvasive and quick risk assessment in infants in the future.

## Availability of supporting data

### Ethics approval and consent to participate

Parents provided written informed consent, and the study was approved by the Regional Ethical Board in Stockholm. The study was conducted in accordance with the standards specified in the 1964 Declaration of Helsinki.

### Consent for publication

Written informed consent was obtained from the models for publication of their image in this manuscript (Figure 1). The consent is held by the author and is available for review by the Editor-in-Chief.

## Abbreviations

DSM-5: Diagnostic and Statistical Manual of Mental Disorders
